# Supplementary material for: Comparative Metabolic Profiling of Grape Skin Tissue along Grapevine Berry Developmental Stages Reveals Systematic Influences of Root Restriction on Skin Metabolome
Source: Int J Mol Sci. 2019 Jan 28;20(3):534. doi: 10.3390/ijms20030534 (PMC6386830; doi:10.3390/ijms20030534)
Supplement: Supplementary file 1 [file ijms-20-00534-s001.zip › Supplementary figures-revision.pdf]

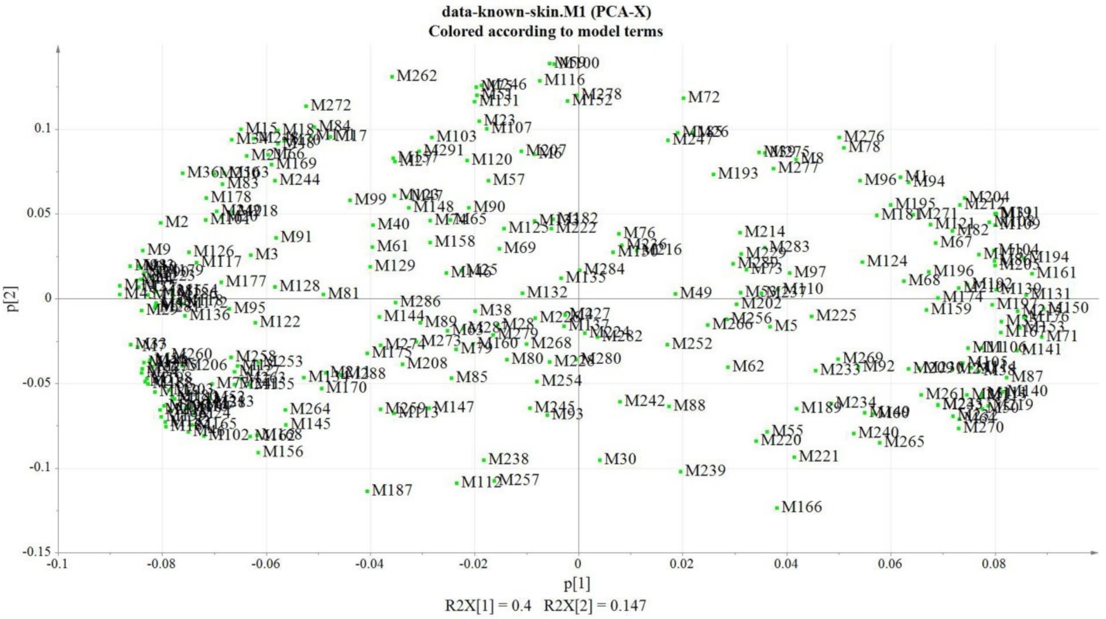

Supplementary Figure S1. Loading scatterplot from the PCA analysis of berry skin in RR and RC cultivation method. Each metabolite referred to Supplementary Table S1.

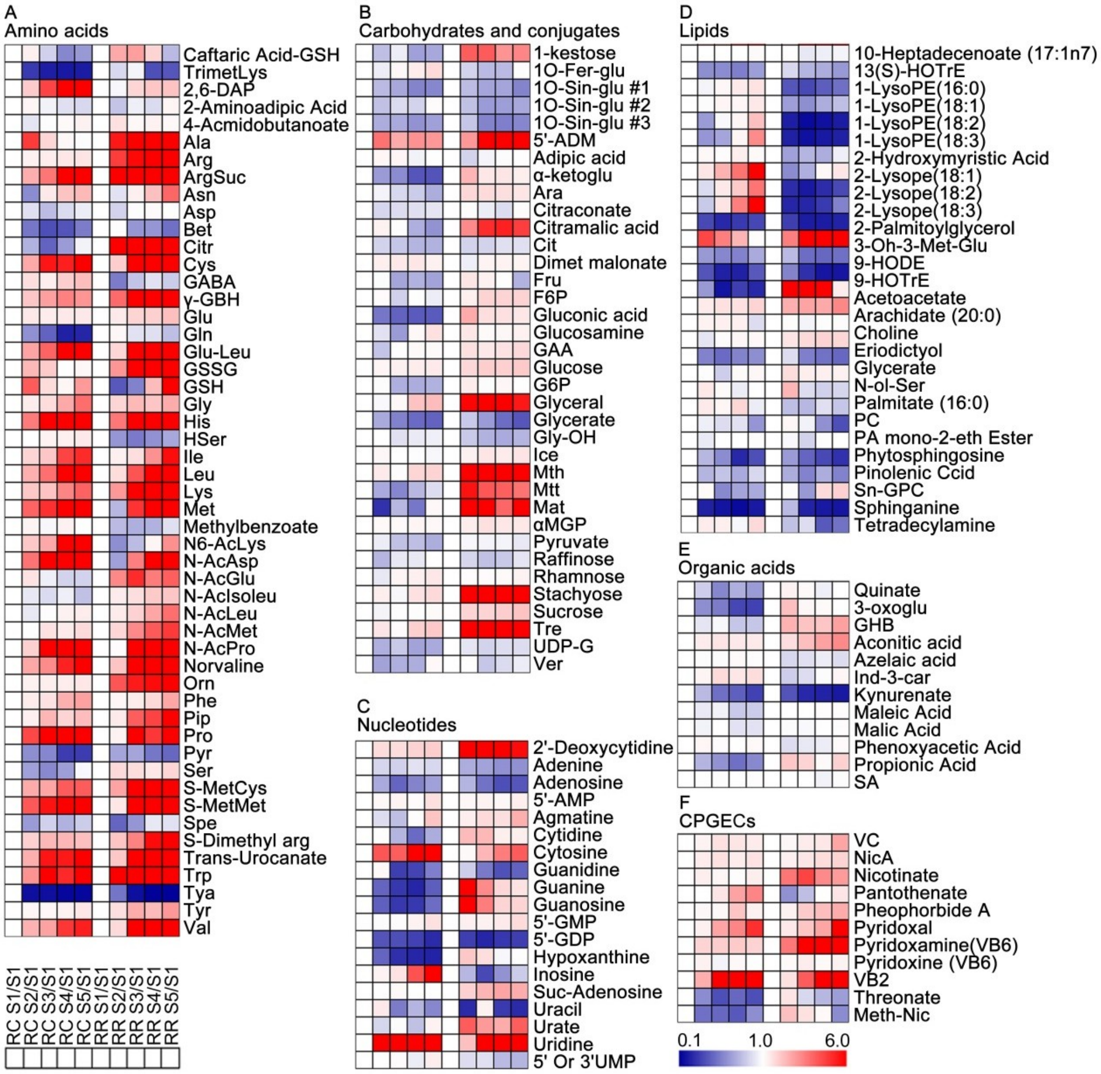

Supplementary Figure S2. Heatmap of the primary metabolite changes in skin tissues of developing berries for each cultivation method as compared with S1 stage. (A) Amino acids and derivatives. (B) Carbohydrates and conjugates. (C) Nucleotides. (D) Lipids. (E) Organic acids. (F) CPGECs. RR and RC are the abbreviations of root restriction and traditional cultivation, respectively. T-test was performed for statistical analysis. Each data was mean value of three biological replicates. The full names of metabolites were attached to Supplementary Table S1.

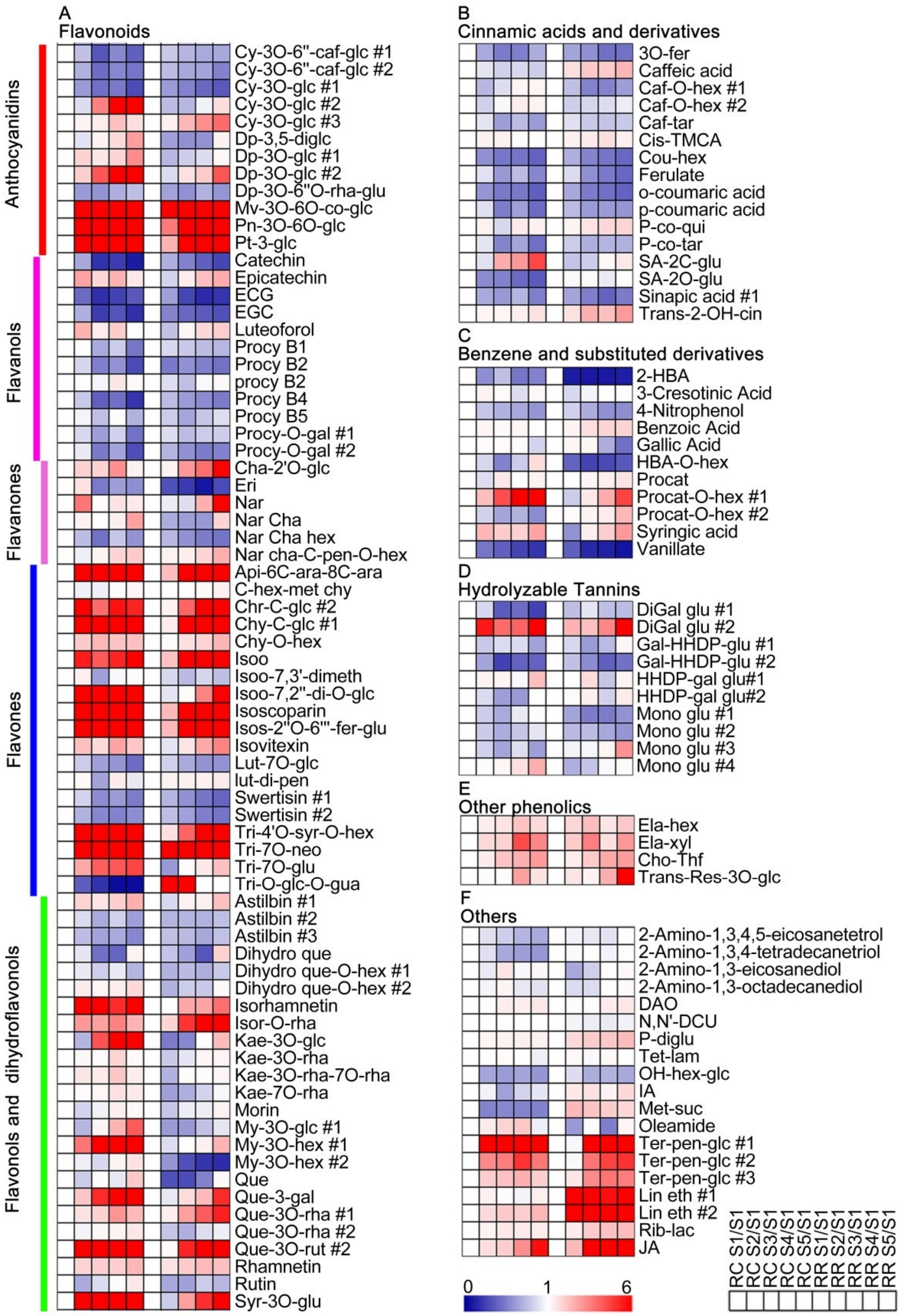

Supplementary Figure S3. Heatmap of the secondary metabolite changes in skin tissues of developing berries for each cultivation method as compared with S1 stage. (A) Flavonoids, including anthocyanins, flavanols, flvanones, flavones and flavonols and dihydraflavonols. (B) Cinnamic acids and derivatives. (C) Benzene and substituted derivatives. (D) Hydrolysable tannins. (E) Other phenolics. (F) Others. RR and RC are the abbreviations of root restriction and traditional cultivation, respectively. T-test was performed for statistical analysis. Each data was mean value of three biological replicates. The full names of metabolites were

attached to Supplementary Table S1.

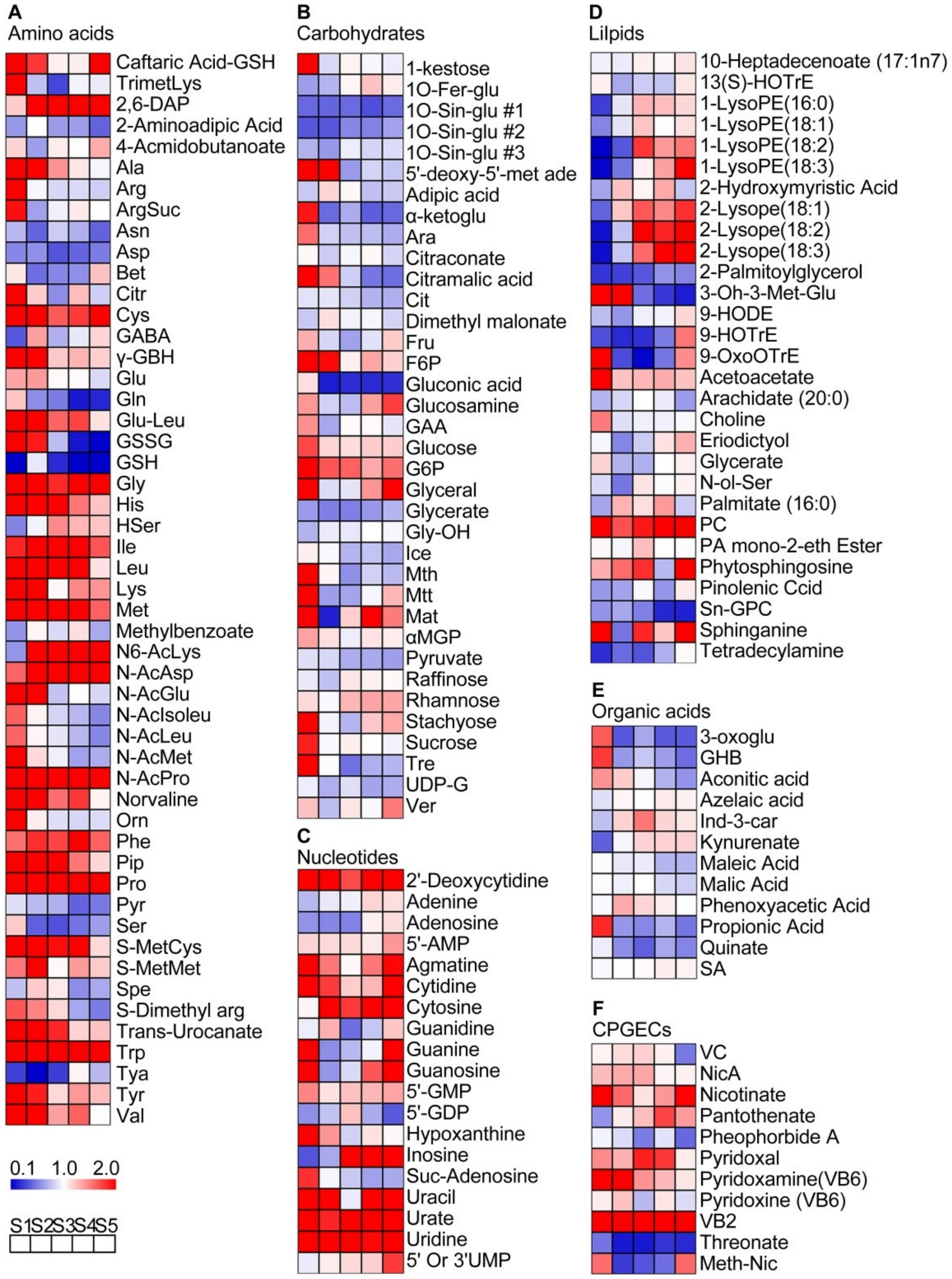

Supplementary Figure S4. Heatmap of the primary metabolites changes in skin tissues of developing berries under RR condition as compared with those in RC (RR vs RC). (A) Amino acids and derivatives. (B) Carbohydrates and conjugates. (C) Nucleotides. (D) Lipids. (E) Organic acids. (F) CPGECs. RR and RC are the abbreviations of root restriction and traditional cultivation, respectively. T-test was performed for statistical analysis. The full names of metabolites were attached to Supplementary Table S1.

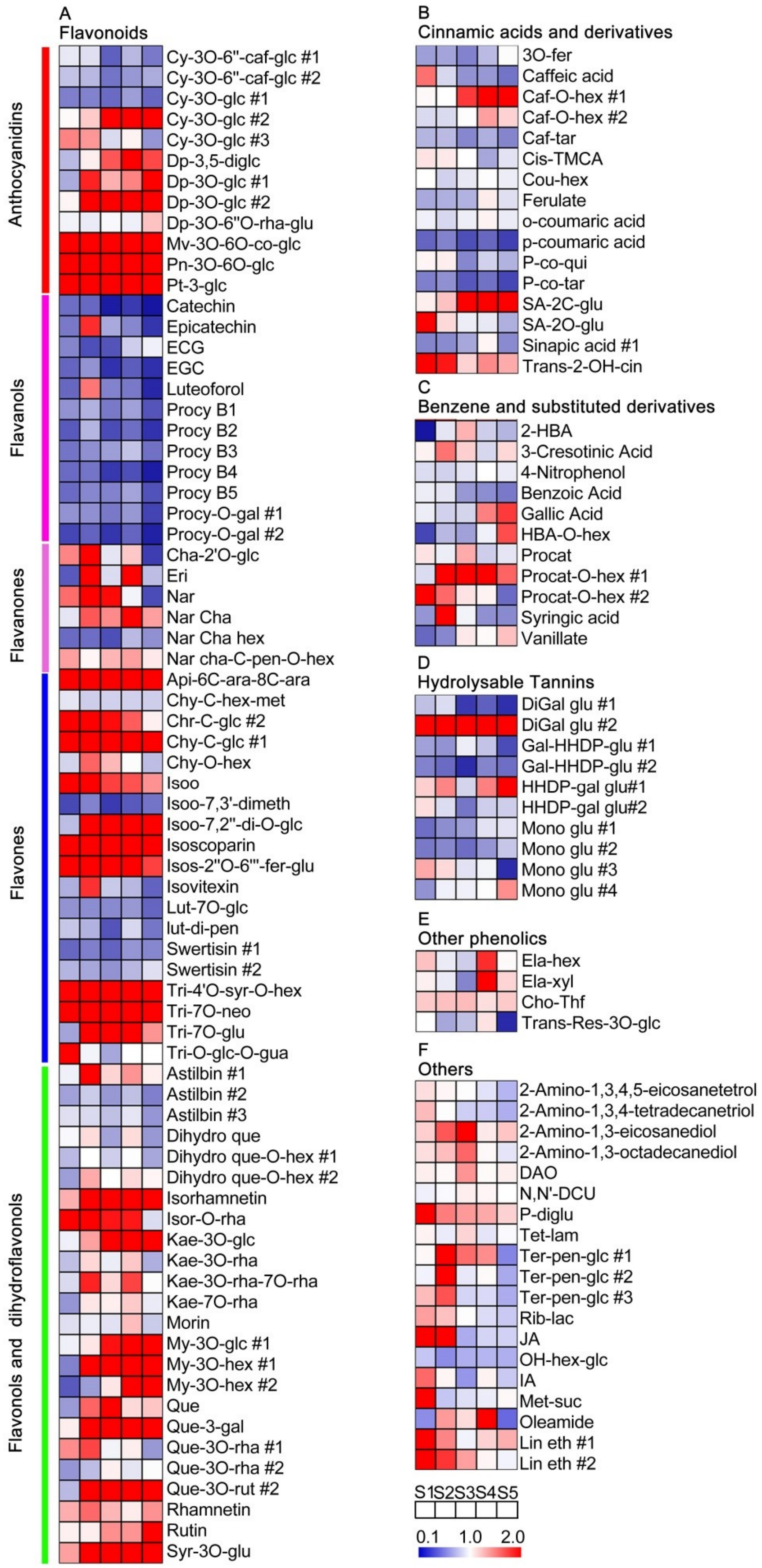

Supplementary Figure S5. Heatmap of the secondary metabolites changes in skin tissues of developing berries under RR condition as compared with those in RC (RR vs RC). (A)

Flavonoids, including anthocyanins, flavanols, flvanones, flavones and flavonols and dihydraflavonols. (B) Cinnamic acids and derivatives. (C) Benzene and substituted derivatives. (D) Hydrolysable tannins. (E) Other phenolics. (F) Others. RR and RC are shorted for root restriction and root control, respectively. T-test was using for statistical analysis. Full metabolites names were referred to Supplementary Table S1.

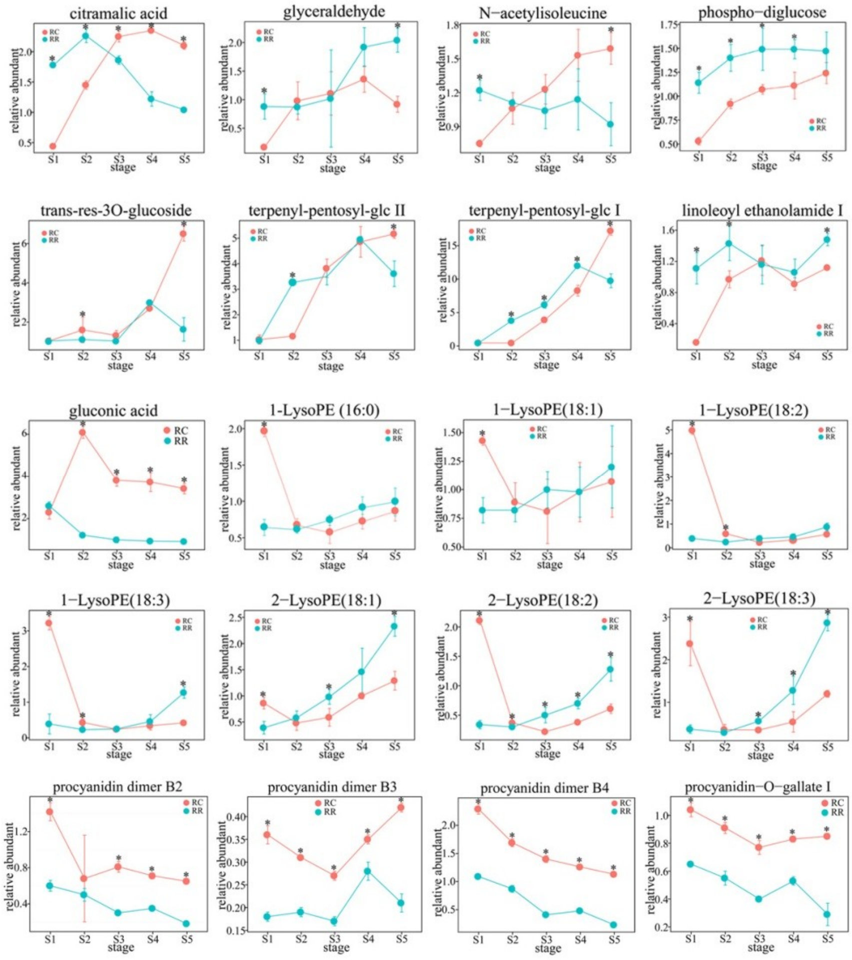

Supplementary Figure S6. Variations of representative metabolites during the developmental stage by Fisher's man analysis. RR and RC are shorted for root restriction and root control, respectively. T-test was using for statistical analysis. The relative levels of metabolites were averaged with three biological replicates.
